# Social bonds, social status and survival in wild baboons: a tale of two sexes

**DOI:** 10.1098/rstb.2019.0621

**Published:** 2020-09-21

**Authors:** Fernando A. Campos, Francisco Villavicencio, Elizabeth A. Archie, Fernando Colchero, Susan C. Alberts

**Affiliations:** 1 Department of Anthropology, University of Texas at San Antonio, San Antonio, TX, USA; 2 Department of Biology, Duke University, Durham, NC, USA; 3 Department of Evolutionary Anthropology, Duke University, Durham, NC, USA; 4 Department of International Health, Bloomberg School of Public Health, Johns Hopkins University, Baltimore, MD, USA; 5 Interdisciplinary Center on Population Dynamics, University of Southern Denmark, Odense, Denmark; 6 Department of Biological Sciences, University of Notre Dame, Notre Dame, IN, USA; 7 Institute of Primate Research, National Museums of Kenya, Nairobi, Kenya; 8 Department of Mathematics and Computer Science, University of Southern Denmark, Odense, Denmark

**Keywords:** Bayesian model, dominance rank, mortality, primates, social relationships, time-varying covariates

## Abstract

People who are more socially integrated or have higher socio-economic status live longer. Recent studies in non-human primates show striking convergences with this human pattern: female primates with more social partners, stronger social bonds or higher dominance rank all lead longer lives. However, it remains unclear whether social environments also predict survival in male non-human primates, as it does in men. This gap persists because, in most primates, males disperse among social groups, resulting in many males who disappear with unknown fate and have unknown dates of birth. We present a Bayesian model to estimate the effects of time-varying social covariates on age-specific adult mortality in both sexes of wild baboons. We compare how the survival trajectories of both sexes are linked to social bonds and social status over the life. We find that, parallel to females, male baboons who are more strongly bonded to females have longer lifespans. However, males with higher dominance rank for their age appear to have shorter lifespans. This finding brings new understanding to the adaptive significance of heterosexual social bonds for male baboons: in addition to protecting the male's offspring from infanticide, these bonds may have direct benefits to males themselves.

This article is part of the theme issue ‘Evolution of the primate ageing process'.

## 1. Background

Sociologists have long known that social integration [1,2] and socio-economic status [3,4] are among the most powerful predictors of mortality risk in humans. In the last decade, similar strong relationships between lifespan and social environments have been revealed in primates [5–13], hyraxes [14], ungulates [15,16], whales [17,18], rodents [19], carnivores [20] and lagomorphs [21]. These findings have enhanced our understanding of the evolution of animal social relationships and uncovered links between sociality and ageing [22].

Evolutionary theories to explain sex differences in lifespan posit that males and females have different optima in tradeoffs between survival and reproduction [23]. Nonetheless, in long-lived iteroparous mammals, empirical and theoretical work indicates that lifespan is the most important component of Darwinian fitness in both males and females [24,25]. In spite of this fact, the relationship between social environments and survival in male mammals remains understudied. In the recent proliferation of studies linking social gradients to survival in wild mammals, the sexes were not differentiated in some cases [8,10], but in others, only females have been studied [5–7,9,11–13,26–28]. This focus on females stems from the fact that, in most social mammals, including most primates, females typically remain in their natal group throughout their lives, while males move in and out of study populations, resulting in individuals with truncated data (unknown dates of birth) and censored data (disappeared with unknown fate). Therefore, it remains unclear to what extent strong social bonds and high social status enhance longevity in male non-human primates, as they do in men [1,2,4,29,30]. Understanding the link between social environments and longevity in males is important: if the male pattern is different to the female pattern, different selection pressures on social behaviour will apply in the two sexes; if the patterns are similar, social relationships for males might have fitness benefits that have previously been overlooked.

Recently, Bayesian estimation methods have been developed to overcome the challenge of estimating age-specific mortality trajectories from datasets with highly censored and truncated data [31]. A key advantage of these methods is that the various sources of uncertainty—including ages of immigrant animals and the fates of animals that disappear—are propagated through to the parameter estimates of the posterior distributions for more reliable inference. However, until now these methods have not incorporated covariates that vary over the life course. Here, we present a Bayesian model that enabled us to include time-varying covariates of social bond strength and social status (dominance rank) in estimating age-specific mortality in wild male and female baboons (*Papio cynocephalus*). We apply this model to an unprecedented dataset spanning 35 years of longitudinal life-history data and fine-grained observations of social environments for 265 adult female and 277 adult male baboons in Amboseli, Kenya. Our methodological advances enable us to compare, for the first time, how the survival trajectories of both males and females are linked to social bonds and social status in a wild non-human primate. Our results contribute to a growing body of evidence that takes advantage of non-human primate studies to shed light on physiological, reproductive and actuarial senescence in humans, and on the evolution of lifespan [31–37].

## 2. Methods

### (a) Study system and subjects

The baboons of the Amboseli basin, southern Kenya (2.667 S, 37.283 E) have been under continuous observation since 1971 [38]. Like most species of baboons, the study subjects live in multi-male, multi-female social groups in which individuals mate and socialize with multiple partners [39]. Amboseli lies in a hybrid zone between two baboon species; study subjects are yellow baboons (*Papio cynocephalus*) that show both historic and recent admixture with anubis baboons (*Papio anubis*) [40–43]. The subjects included *n* = 542 adult baboons—265 females and 277 males—that resided in study groups between January 1984 and December 2018, and were individually recognized and followed on a near-daily basis. To focus on adult survival, we excluded data prior to age 5 years for females (when menarche most often occurs) and 7 years for males (the earliest age in which males attain a dominance rank among other adult males [44]). Birth dates were known to within a few days for 232 of 265 females and 108 of 277 males (62.7% of subjects). For the remaining individuals, ages were estimated by experienced observers and bracketed with maximum and minimum estimates (162 immigrant males, 23 females and 5 males born before observations on a group began, and 10 females and 2 males with more than a few days of uncertainty in the birth date). This estimated age then informed a process of modelling individual age, as described below.

### (b) Social bonds

We included time-varying measures of social bond strength in our models of age-specific mortality. To make these values comparable over the life courses of different individuals, each individual's covariates were measured during each 1-year age class lasting from one birthday to the next. A year of life could be incomplete on the left if the individual entered the study between birthdays, either by reaching adulthood, immigrating, or at the onset of observations on their study group. A year of life could be incomplete on the right by death or censoring (i.e. disappearance from the population, or reaching the end of the study period).

We quantified each individual's social bond strength with male and female social partners separately using an approach modified from previous studies [6,45,46]. We first calculated a ‘dyadic sociality index' (DSI) that measures bond strength between pairs of adult animals in each group in each year of life based on dyadic grooming behaviour (see electronic supplementary material). The DSI is calculated from the relative frequencies with which each dyad exchanged grooming, corrected for observer effort (i.e. corrected for a bias that results from differences in social group sizes; see electronic supplementary material, figures S1 and S2). This initial DSI value for a given dyad, therefore, represents the ‘strength' of their grooming relationship relative to all other dyads in the population, controlling for observer effort.

After obtaining these initial DSI values, we standardized the DSI values of all adult dyads in the population during the given year of life, separately for female–female dyads and female–male dyads, using *z*-score transformations. This standardization enabled us to define DSI as the dyad's bond strength relative to all other grooming dyads of that type that were present at the same time in the study population. Notably, female–female and female–male dyads exhibit qualitatively similar rates of grooming, and one bond type is not substantially stronger than the other in a given life year (electronic supplementary material, figure S3). By standardizing, we removed the effects of population-wide fluctuations in grooming rates due to environmental or demographic factors that varied among life years (electronic supplementary material, figure S4). For female subjects, we defined DSI_F_ as the mean DSI value between the focal female and her top three female grooming partners, and DSI_M_ as the mean DSI value between the focal female and her top three male grooming partners. For male subjects, we only calculated DSI_F_, as adult male–male dyads do not regularly groom each other in this population.

### (c) Social status

We used time-varying measures of social status in our models of age-specific mortality, by measuring each individual's dominance rank during each 1-year age class. Adult females and males were ranked separately, based on observations of decided dyadic agonistic interactions between two adult animals of the same sex, collected via representative interaction sampling. Decided interactions were those in which one animal gave only aggressive or neutral behaviours while the other animal gave only submissive behaviours. Wins and losses for each month were compiled in a pairwise interaction matrix, and ordinal ranks for that month were determined by minimizing the number of wins below the diagonal. We converted these ordinal ranks to proportional ranks that expressed the proportion of adult animals of the same sex in the same group that were dominated by the focal animal. Proportional ranks ranged from 0 (lowest-ranking) to 1 (highest-ranking). In the mortality models, we used the mean of each individual's monthly dominance ranks in each year of life.

### (d) Models of age-specific mortality

We extended the model developed by Colchero and colleagues [31,47] to make inferences on age-specific mortality of male and female baboons when age information is uncertain and when males disperse outside the study population. The model requires estimating dispersal outside the study population, which we call out-migration (i.e. permanent departure from the study groups), because many individuals were still alive when they were last seen and thus they could have either died or left the study population to join other groups. In our dataset of 265 females and 277 males, departure from the study occurred by death for 129 females (48.7%) and 41 males (14.8%), by right-censoring (i.e. alive at the end of observation period) for 136 females (51.3%) and 129 males (46.6%), and by disappearance with unknown fate for 0 females and 107 males (38.6%).

Our model of adult mortality is conditioned on reaching maturity, age *α* (5 for females and 7 for males). Because some animals (e.g. immigrants) had uncertain ages, to define the survival intervals, we defined random variables for ages at death *X*, for ages at natal out-migration *Y*, and for ages at immigrant out-migration *Z*, with realizations *x, y, z* ≥ *0*, where *x* = (age at death − *α*), *y* = (age at natal out-migration − *α*), and *z* = (age at immigrant out-migration − *α*). Natal out-migration refers to the first out-migration of individuals who were in the study population when they reached age at maturity, whereas immigrant out-migration refers to first, second, and subsequent out-migrations of individuals who entered the study population after reaching age at maturity (immigrants). We also defined a random variable indicator *O* for the out-migration state, which gets a value of 1 if the individual had out-migrated and 0 otherwise.

To model mortality, we used the Gompertz law [48], which fits well the adult mortality of baboons [32]. The mortality risk (or hazard) of the Gompertz model is usually expressed as

$$\mu (x)=a\;e^{bx},$$

where *x* refers to age, and *a*, *b* > 0 are the baseline mortality and rate parameters, respectively. One difference between this and previous implementations is that here we modelled natal and immigrant out-migration conditioned on the out-migration state *o_ij_* = 0, 1, where *i* = 1, …, *n* and *j* = 1, 2 is the index for natal or immigrant out-migration. The out-migration state has Bernoulli probability. An individual with *o_ij_* = 1 contributes to the estimation of the gamma-distributed ages at out-migration. For individuals with unknown state, the model samples the out-migration state at every iteration, where the conditional posterior for out-migration state incorporates the likelihood that the individual died against the likelihood that they out-migrated (see extended model description in electronic supplementary material).

Following the implementation in Colchero *et al.* [31], we used an agent-based model to estimate the priors for the out-migration parameters, with the addition of priors for the probability of out-migration. The agent-based model used empirical data on known ages at natal dispersal and higher-order dispersal in male baboons, as well as data on the number of study groups and the number of surrounding non-study groups to which out-migrating males could disperse, to estimate the priors for out-migration and death for unknown-fate individuals.

Unlike previous implementations of the model [31,47], our extension enables the inclusion of time-varying covariates for survival, crucial for testing the effects of social bond strength and social status on survival. The covariates change during the life-course of most individuals. We standardized all covariates by sex and age to remove systematic differences in social bond strength and status among animals of different ages. For example, in males, bond strength with females and social status tend to peak in early adulthood and decline later in life [49] (electronic supplementary material, figure S5). Accordingly, the covariate values represent deviations from sex- and age-typical values of social bond strength and status.

For the time-varying covariates, the hazard function is given by

$$h(x|w_{x})=\mu (x)\;e^{\kappa \cdot w_{x}},$$

where *****w**_x_*** ∈ $R^{3}$ is a vector of time-varying covariates at age *x*, and ***κ*** ∈ $R^{3}$ is a proportional hazards vector to be estimated. The cumulative hazard is approximated as

$$H(x|W)\approx \overset{x}{\underset{t=0}{\sum }}\mu (t)\;e^{\kappa \cdot w_{t}},$$

where ***W =*** [***w*_0_**, ***w*_1_**, … , ***w_x_***] is a matrix of the time-varying covariates for all observed ages. Then, the cumulative survival function is given by

$$S(x|W)=e^{-H(x|W)},$$

while the probability density function of ages at death is

$$f(x|W)=h(x|w_{x})\;S(x|W).$$

For all mortality parameters, including the proportional hazards parameters, we used vague priors. We sampled all parameters and unknown out-migration states using Metropolis-Hastings [50,51] sampling within a Markov chain Monte Carlo (MCMC). To assess convergence, we ran eight parallel chains with 5000 iterations and an initial burn-in sequence of 1000, and calculated potential scale reduction factor $\left(\hat{R}\right)$ [52].

### (e) Data imputation

**Figure 1.**
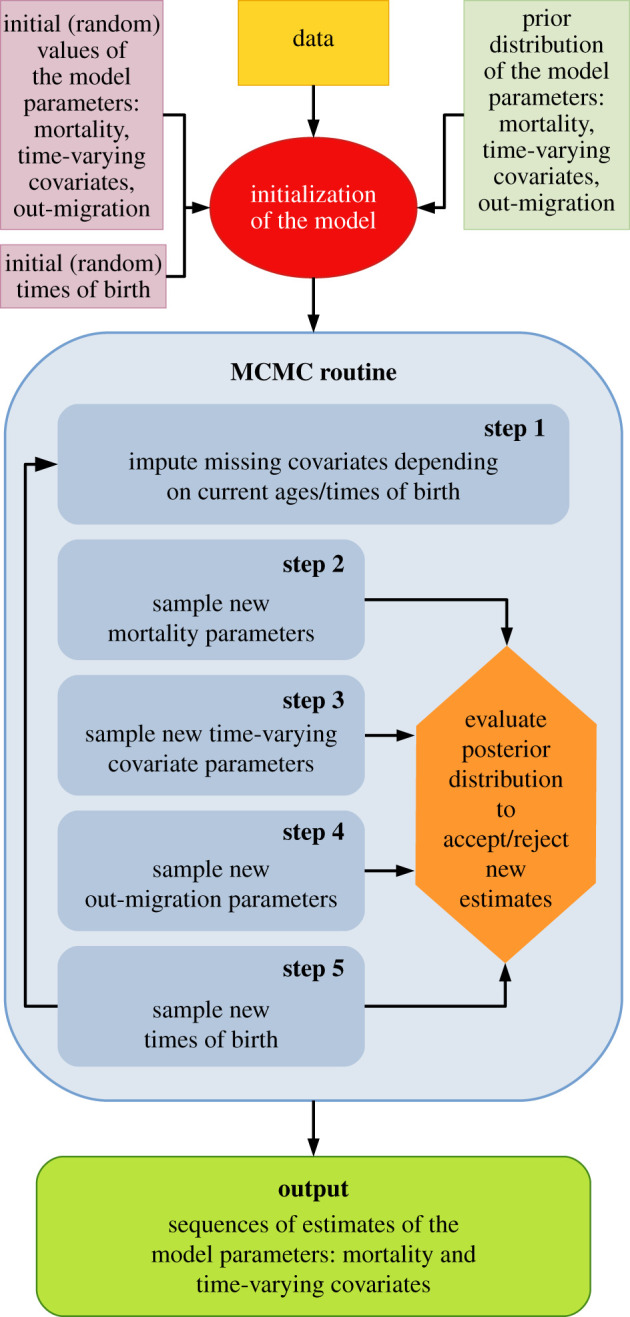
Workflow of the Bayesian model and the MCMC routine. (Online version in colour.)

Data imputation was necessary because measurements of DSI_F_, DSI_M_ or proportional dominance rank were missing for some individual-years of observation; for example, the covariate values of immigrant adult males were unknown prior to their entry into a study group (electronic supplementary material, figure S6). Missing covariates were imputed randomly at each iteration of the MCMC, for any missing covariate values between age at maturity (five for females and seven for males) and the individual's oldest observed age. Specifically, for each of the three covariates, we calculated the age- and sex-specific mean, standard deviation, minimum and maximum values of the observed covariates using the imputed ages/times of birth in the current iteration of the MCMC. Next, new values for the missing covariates were randomly drawn from a truncated normal distribution defined by the corresponding age- and sex-specific means, standard deviations, minimums and maximums. For example, if for a given iteration, age and proportional rank were observed for 100 males, but missing for 50 males, we computed the mean, standard deviation, minimum and maximum of the 100 observed values, and then imputed proportional rank for the other 50 males by drawing values from a truncated normal distribution with the corresponding parameters. For the few cases (occurring in old age) in which all subjects (or all but a single subject) had missing covariates for a given age and sex, missing covariates were imputed randomly independently of age. Figure 1 presents a workflow of the Bayesian model, including the data imputation.

To verify that the results of the survival analysis were not affected by this data imputation method—which we refer to as the ‘full model'—we also applied three alternate methods of data imputation and compared the results. These three alternative approaches involved running the model with subsets of the data that included complete information on social bond strength with females (54 males, 184 females) or social status (75 males, 187 females), and filling data gaps using linear interpolation. We describe these procedures in the electronic supplementary material. In addition, all results are reproducible from the R code and data available in Dryad (https://doi.org/10.5061/dryad.kh189322b).

## 3. Results

The model produced stable estimates of all parameters (electronic supplementary material, figures S7 and S8; $\hat{R}$ values in electronic supplementary material, table S1). This result justified our use of these estimated trajectories of survivorship to measure the relationship between survival and social environments over the adult life course (electronic supplementary material, figure S9). Furthermore, all data imputation methods produced similar patterns for the effects of DSI_M_, DSI_F_ and dominance rank on survival (electronic supplementary material, figure S10).

**Figure 2.**
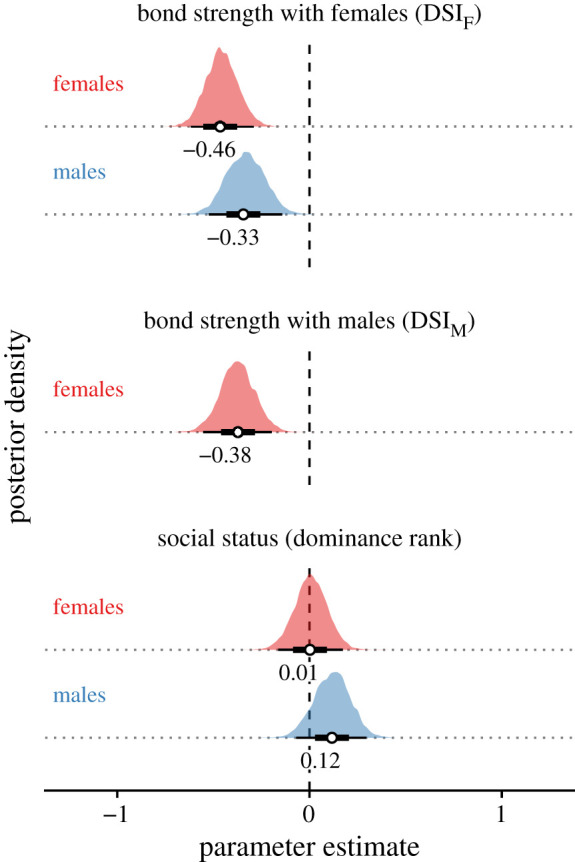
Posterior densities of the *κ* parameters that measure the effects of social variables on female and male survival. White points show medians; thick black bars show 68% credible intervals; thinner black bars show 95% credible intervals of the posterior distributions. Negative effect sizes indicate that higher values of a predictor variable were associated with lower mortality risk. (Online version in colour.)

**Figure 3.**
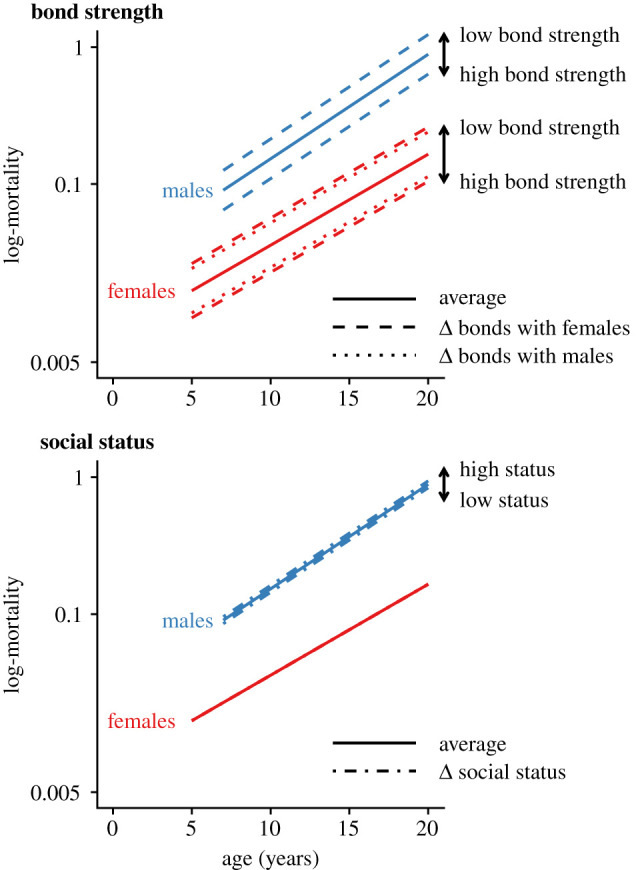
Predicted effects of changes in social bond strength and dominance rank on log-mortality. The continuous lines represent the log-hazard of the Gompertz model based on the baseline parameters estimated by the Bayesian model. Lines labelled ‘low bond strength' and ‘high bond strength’ show one standard deviation of increase or decrease in DSI_F_ or DSI_M_ relative to the population mean standardized for age (solid line), and how that translates to log-mortality. The lines labelled ‘high status’ and ‘low status' show a change of one standard deviation of proportional dominance rank relative to the population mean standardized for age (solid line). (Online version in colour.)

Social bond strength strongly predicted survival for adult baboons of both sexes (figures 2 and 3; electronic supplementary material, table S1). For males, an increase of one standard deviation in DSI_F_ translated to a reduction of approximately 28% in the mortality hazard for any given age. For females, increases of one standard deviation in DSI_F_ and DSI_M_ were associated with stronger reductions than in males: 37% and 31% reductions in mortality hazard for any given age.

Higher social status in adult male baboons was associated with a reduction in survival, although the 95% (but not the 68%) credible interval included zero (figures 2 and 3; electronic supplementary material, table S1). For males, an increase of one standard deviation in proportional dominance rank for their age was associated with an increase of approximately 13% in the mortality hazard for any given age. We saw no relationship between female social status and survival (figure 2).

## 4. Discussion

### (a) Social bonds and sex-specific survival

Using novel methods for analyzing datasets with missing and uncertain data, we found that both male and female baboons who had stronger social bonds had improved survival. Male baboons with strong social bonds to females experienced a 28% reduction in the mortality hazard for any given age compared to males with weak social bonds to females. This is the first evidence in a wild non-human primate that males, like females, show a strong link between social bond strength and survival over the natural lifespan. This finding brings new understanding to the potential adaptive significance of heterosexual social bonds for male baboons, especially in light of the absence of enduring same-sex social bonds among male baboons in our population. The benefits of ‘friendships' for male baboons and other social primates have previously been interpreted in terms of male reproductive advantages, such as securing future mating opportunities with female friends and protecting the male's offspring from infanticide and harassment [53–55]. Our findings demonstrate that an additional potential benefit of ‘friendships' with females is extended lifespan.

Lifespan is no less important for male fitness than for female fitness. Indeed, for long-lived species, variation in lifespan has a bigger effect on fitness than variation in fertility, even in societies in which male mating is highly competitive and is concentrated during the prime years of life [24,25]. The common misconception that lifespan ‘matters less’ for males in competitive societies may stem from the idea that most of the variation in lifespan is accounted for by males who live past the prime reproductive age; this is incorrect. For example, in the Amboseli baboons, half of males who reach adulthood die *during* the prime reproductive years of 8–13 years of age [56]. The same is true for many other species with competitive mating (e.g. 57,58 for multiple species of ungulates). Thus, an extension in lifespan for male primates will often mean an increase in survival through their prime reproductive years, as well as an enabling of reproduction into old age. For this reason, understanding sources of variation in male lifespan provides us with essential insight into traits that are likely to be under selection.

Our findings for adult female survival add to a growing body of evidence that links social bonds with female longevity in various populations of non-human primates [5–7,9,11]. They also recapitulate previous work in our study population, which used a different metric of social bonds called ‘social connectedness', a measure of normalized grooming frequency with all partners of a given sex [6]. By focusing on the strength of a female's most important social bonds (DSI_F_) in this study, we employ a metric that more closely parallels measures used in human studies [2], and one that is similar to several other nonhuman primate studies [5,9,11]. In this study, social bonds with females (DSI_F_) were a stronger predictor of female survival than social bonds with males (DSI_M_), whereas in our previous study the stronger predictor was social connectedness with males [6]. In spite of these differences—which might be driven by the use of a different metric—the directions of the effects were consistent and had strong empirical support in both studies.

What mechanisms underlie the links between social bonds and survival [59,60], and do those mechanisms differ between the sexes? In female cercopithecines, same-sex social bonds may benefit females by increasing tolerance during competition over limited resources, facilitating the acquisition of high dominance rank, and strengthening alliances [59,60]. Opposite-sex social bonds may benefit females by providing protection against harassment, predation and infanticide [55,61]. By contrast, much less attention has been paid to mechanisms by which opposite-sex social bonds enhance survival for male primates. Grooming relationships with either sex could provide direct health and survival benefits in the form of reduced ectoparasite burden [62], improved vigilance for predators [63], and environmental buffering or stability [64,65].

Through such mechanisms, both sexes may experience positive effects of affiliative behaviour on neuroendocrine signalling [66]. In female rhesus macaques (*Macaca mulatta*), grooming interactions lead to reduced heart rate [67]; in female chacma baboons (*Papio ursinus*) and female rhesus macaques, aspects of social bonds predict lower levels of glucocorticoid metabolites in faeces, indicating reduced HPA axis activation in response to stressors [68–70]; and in eastern chimpanzees (*Pan troglodytes schweinfurthii*) of both sexes, social support from strongly bonded partners reduces levels of urinary glucocorticoids [71]. Furthermore, male barbary macaques that form close ‘friendships' with other males show attenuated patterns of glucocorticoid secretion, suggesting that they are buffered against social and environmental stressors [72]. While none of these studies focused specifically on health or survival benefits that males may gain from forming bonds with females, they suggest that reduction of HPA axis activity may be a proximate mechanism that connects greater social bonds with improved health and survival outcomes, because chronically elevated glucocorticoids have been associated with a wide range of pathologies in both humans and other animals [73–76].

Importantly, a simple correlation, rather than a causal relationship, cannot be ruled out. Healthier individuals, in better condition, may be more likely to live long lives, more likely to maintain strong social bonds, and more likely to achieve high social status. In this scenario, longer lifespans are not a direct benefit of social bonds, but instead social bonds and longer lifespans both flow from better physical condition. Future analyses could make progress on this question by using longer-term or time-lagged measures of sociality to predict survival. However, in human studies, the evidence for a direct causal effect of social bonds on survival is considered strong, although causality is difficult to establish unambiguously [2]. Causal inference modelling, an approach that has attracted growing interest in human population studies, can provide powerful insights into this problem, and represents a strong future direction for studies in natural populations.

### (b) Social status and sex-specific survival

Our findings provide new insights into sex differences in the relationship between social status and lifespan. In humans, the powerful and remarkably consistent link between socioeconomic status and lifespan in both sexes is well established [3,4]. Many social mammals also compete for high social status, but most research in this area has focused on reproductive advantages associated with high social status. The relatively few studies that have examined the relationship between social status and survival in non-human primates paint a less straightforward picture than in humans [22]. Among female primates, high social status is associated with a survival advantage in some populations of macaques and baboons [5,12,13] but not in other primates [6,9,26,27]. As in a previous study with this population [6], we found no direct relationship between social status and survival in female baboons. The evolution of competition for high social status among female baboons is therefore likely to be determined by the nutritional and reproductive advantages of high social status [77] rather than by variation in survival. However, female social status may have an indirect effect on survival because a females' social bonds with males are predicted by female dominance rank, with higher ranking females having greater ‘social connectedness' to males [6].

In contrast to studies in humans, we found that males who maintain higher social status for their age tend to have shorter lifespans, although the evidence was weaker than for the relationship between social bonds and survival. Male baboons compete intensely to attain high social status, and high status confers large reproductive advantages on males [49]. Thus, our results point to a possible trade-off between lifespan and reproductive success in male baboons that may shape the evolution of male–male competition for social status. Several other lines of evidence support such a tradeoff in male baboons. First, the highest-ranking male baboons in a social group have elevated levels of both testosterone and glucocorticoids [78], both of which have immunosuppressive effects that can compromise health and survival, but may be critical for mediating reproduction and responding to social challenges in male primates [79,80]. Second, high social status is associated with faster epigenetic ageing in male baboons in Amboseli [81]. Third, studies of gene expression indicate that high-ranking males in Amboseli show increased expression of inflammation-related genes relative to low-ranking males, suggesting that they may disproportionately incur costs associated with higher rates or intensities of acute inflammatory responses [82]. In humans, both sexes show gradients in health related to social status, but some evidence suggests that men's health may be more sensitive to differences in social status than women's health [83]. This pattern appears to have echoes in our population of wild baboons, with the important difference that social status does not directly predict female survival. Further research on the physiological mechanisms that link social bonds and social status with health and survival in baboons and other social mammals will be crucial for illuminating the evolutionary significance of these parallels and contrasts between baboons and humans.

## Supplementary Material

Supplementary methods, figures, and table

## Data accessibility

Data and R code for reproducing the analysis are available from the Dryad Digital Repository: https://doi.org/10.5061/dryad.kh189322b [84].
