# Supplementary material for: Social bonds, social status and survival in wild baboons: a tale of two sexes
Source: Philos Trans R Soc Lond B Biol Sci. 2020 Sep 21;375(1811):20190621. doi: 10.1098/rstb.2019.0621 (PMC7540948; doi:10.1098/rstb.2019.0621)
Supplement: Supplementary methods, figures, and table [file rstb20190621supp1.pdf]

Supplementary materials for
Social bonds, social status, and survival in wild baboons: a tale of two sexes

Fernando A. Campos†, Francisco Villavicencio†, Elizabeth A. Archie, Fernando Colchero, and Susan C. Alberts*

† These authors contributed equally.
* Corresponding author: alberts@duke.edu.

Grooming data and bias correction

Beginning in 1984, grooming interactions were recorded by highly experienced observers, typically during 5-hour-long observation periods that occurred several days each week with each baboon social group. Up to seven baboon social groups were observed contemporaneously over the 35 years spanned by the study. The grooming data were collected using an approach that we refer to as "representative interaction sampling" in which an observer moves systematically through the group while carrying out 10-minute focal animal samples according to a predefined, randomized list of subjects, and simultaneously recording all grooming interactions in their line of sight. This approach has been previously validated to ensure that it results in even representation of all group members and patterns of interaction frequencies and partner preferences that show close correspondence with those derived from focal animal sampling alone [1].

A bias in dyadic grooming rates resulting from differences in social group sizes must be corrected when using representative interaction sampling data from different groups. This bias occurs because, in larger groups, observer effort is more thinly distributed across group members (i.e. the ratio of animals to observers is larger) (**Fig. S1A**). Hence, we are less likely to observe a grooming interaction between a given pair of animals in a large group compared to a small group. To address this bias, we regressed the logarithm of observer effort (number of focal samples per female per day of observation on the group) on the logarithm of the dyadic grooming rate (number of interactions per day of co-residence for the dyad), and used the residuals as our initial measure of DSI. This initial DSI value for a given dyad therefore represents the 'strength' of their grooming relationship relative to all other dyads in the population, controlling for observer effort (**Fig. S1B**). The original dyadic grooming rates (log transformed) are strongly correlated with the DSI values that are corrected for observer effort (Spearman correlation coefficient = 0.92, **Fig. S2**).

Bayesian inference on age-specific mortality

The basic inference model for age-specific survival is based on the method proposed by Colchero et al. [2] and Barthold et al. [3]. This model enabled us to make inferences on age-specific survival (or mortality) and on the age at out-migration, namely the age at which individuals permanently leave the study population. The model is particularly appropriate for primate studies that monitor individuals continuously within a study population and when individuals of one or both sexes can potentially out-migrate, while other individuals originating from surrounding groups join the study population (in-migration).

We define a random variable X for adult ages at death, with observations $x \geq 0$. Note that $x = 0$ refers to the age at maturity (5 for females and 7 for males). The model requires defining a hazards rate or mortality function, given here by the Gompertz law [4], of the form

$$\mu(x) = a\, e^{bx}, \tag{1}$$

where $a, b > 0$ are parameters to be estimated. From the mortality model in **Eq. (1)**, the corresponding survival function can be calculated as $S(x) = \exp\left[-\int_0^x \mu(t)dt\right]$, while the probability density function of ages at death is given by $f(x) = \mu(x)\, S(x)$ for $x \geq 0$.

Male baboons often leave their natal groups to join other neighboring groups, which is commonly identified as "natal dispersal". They can then disperse subsequent times, undergoing "secondary dispersal". Although dispersal within monitored groups (i.e. those belonging to the study population) does not affect the estimation of mortality, the fate of individuals that permanently leave the study population can be confounded as a possible death. We identify the process of permanently leaving the study area as "out-migration", which we classify as natal and immigrant out-migration, the first for natal and the second for secondary dispersals to groups that do not belong to the study population. It is particularly important to account for out-migration in our model since not all out-migrations are detected, and therefore the fate of a number of individuals is unknown after their last detection. For these individuals we define a latent (unknown) out-migration state at the time they were last detected, given by the random variable indicator $O,$ with observations $o_{ij} = 0, 1$, where $o_{ij} = 1$ if individual i out-migrated and $o_{ij} = 0$ otherwise, and where $j = 1$ identifies natal out-migration and $j = 2$ immigrant out-migration. For known out-migrations (i.e. recorded as having emigrated) we automatically assign $o_{ij} = 1$. The model therefore estimates the probability of out-migration π_j as a Bernoulli process $O_{ij} \sim \text{Bern}(\pi_j)$. Those assigned as undergoing out-migration as well as known emigrants and immigrants contribute to the estimation of the distribution of ages at out-migration. Here, we define a gamma-distributed random variable V for ages at out-migration, with realizations $v \geq 0$, where $V_j \mid O_j = 1 \sim \text{Gam}(\gamma_{j1}, \gamma_{j2})$, and where $\gamma_{j1}, \gamma_{j2} > 0$ are parameters to be estimated with j defined as above. The probability density function for the gamma distribution is $g_V\left(v - v_j \mid \gamma_{j1}, \gamma_{j2}\right)$, with $v \geq 0$ and where v_j is the minimum age at natal $(j = 1)$ or immigrant $(j = 2)$ out-migration.

In addition, since not all individuals have known birth dates, the model samples the unknown times of birth b_i to estimate the ages at last detection $x_{li} = t_{li} - b_i$, where t_{li} is the time of last detection for individual i. Let $\boldsymbol{\theta} = (a, b)$ denote the vector of mortality parameters. Then, the likelihood is defined as

$$p\left(x_{li} \middle| \boldsymbol{\theta}, \gamma_{j1}, \gamma_{j2}, \pi_j, o_{ij}\right) = \begin{cases} \dfrac{f(x_{li})}{S(x_{fi})}\,(1 - \pi_j) & \text{if } o_{ij} = 0 \\ \dfrac{S(x_{li})}{S(x_{fi})}\, g_V\left(x_{li} - v_j\right) \pi_j & \text{if } o_{ij} = 1 \end{cases} \tag{2}$$

where x_{fi} is the age at first detection for individual i, given by $x_{fi} = t_{fi} - b_i$, with t_{fi} as the corresponding time of first detection. In other words, individuals with $o_{ij} = 0$ are assumed to have died shortly after the last detection, while those with $o_{ij} = 1$ are censored and contribute to the estimation of the distribution of ages at out-migration. The full Bayesian posterior is then given by

$$\begin{aligned} p\left(\boldsymbol{\theta}, \boldsymbol{\gamma}_1, \boldsymbol{\gamma}_2, \boldsymbol{\pi}, \boldsymbol{b}_u, \boldsymbol{o}_u, \boldsymbol{v}_{u1}, \boldsymbol{v}_{u2} \middle| \boldsymbol{b}_k, \boldsymbol{o}_k, \boldsymbol{t}_f, \boldsymbol{t}_l\right) \quad \propto \quad & p(\boldsymbol{x}_l \mid \boldsymbol{\theta}, \boldsymbol{\gamma}_1, \boldsymbol{\gamma}_2, \boldsymbol{\pi}, \boldsymbol{o}) \\ & \times p(\boldsymbol{\theta})\, p(\boldsymbol{\gamma}_1)\, p(\boldsymbol{\gamma}_2)\, p(\boldsymbol{\pi}), \end{aligned} \tag{3}$$

where the first term on the right-hand-side of **Eq. (3)** is the likelihood in **Eq. (2)**, and the following terms are the priors for the mortality and out-migration parameters. The term $\boldsymbol{\pi} = (\pi_1, \pi_2)$ is the vector of probabilities of out-migration, whereas $\boldsymbol{\gamma_j} = (\gamma_{j1}, \gamma_{j2})$ for $j = 1, 2$. The subscripts u and k refer, respectively, to unknown and known birth dates $(\boldsymbol{b})$, out-migration states $(\boldsymbol{o})$, and ages at out-migration ($\boldsymbol{v}_{u1}$ and $\boldsymbol{v}_{u2}$ denote the unknown ages at natal and immigrant out-migration, respectively). The vectors $\boldsymbol{t}_f$ and $\boldsymbol{t}_l$ are the times of first and last detection of all the individuals in the study population.

Alternative data imputation methods

In addition to the full model described in the main text, which used random imputation to fill missing covariates, we ran parallel analyses with three alternative data imputation methods to verify that our conclusions were not affected by the selected method. The results of these analyses are displayed in **Fig. S10** and show that for all methods of data imputation, we obtained the same signal of the effect of the three time-varying covariates on baboon survival. The three alternative methods are the following.

1) "Complete rank data": We ran the model with the subset of individuals observed for the first time no later than one year after reaching age at maturity, and with proportional dominance rank measured every year for the full observed lifespan (i.e. if an individual is observed for 5 years but rank was not recorded the third year, it was excluded from the subset). In this test, dominance rank was never imputed, whereas missing DSI_F and DSI_M were imputed as in the full model. The sample size was reduced by 52% to 187 females and 75 males.
2) "Complete DSI_F data": We ran the model with the subset of individuals observed for the first time no later than one year after reaching age at maturity, and with DSI_F measured every year for the full observed lifespan. In this test, DSI_F was never imputed, whereas missing DSI_M and proportional rank were imputed as in the full model. The sample size was reduced by 56% to 184 females and 54 males.
3) "Linear interpolation": Before running the model, we imputed missing covariates over gaps of one or two years assuming a linear trend. For example, if an individual was observed for six years but DSI_M was missing the third and fourth year, we imputed values for those two years assuming a linear trend between the observed DSI_M on the second and fifth years. The remaining missing covariates were imputed randomly at each iteration as in the full model. The full data set of 542 individuals was used in this test, but the method is more restrictive than the full model because fewer missing covariates are imputed randomly at each iteration.

These three data imputation methods, as well as the results from the full model, some diagnostic plots and the values in **Table S1**, are reproducible from the data and R code available in Dryad (https://doi.org/10.5061/dryad.kh189322b)

References

1. Archie EA, Tung J, Clark M, Altmann J, Alberts SC. 2014 Social affiliation matters: both same-sex and opposite-sex relationships predict survival in wild female baboons. *Proceedings of the Royal Society B: Biological Sciences* **281**, 20141261. (doi:10.1098/rspb.2014.1261)

2. Colchero F *et al.* 2016 The emergence of longevous populations. *PNAS* **113**, E7681–E7690. (doi:10.1073/pnas.1612191113)

3. Barthold JA, Packer C, Loveridge AJ, Macdonald DW, Colchero F. 2016 Dead or gone? Bayesian inference on mortality for the dispersing sex. *Ecology and Evolution* **6**, 4910–4923. (doi:10.1002/ece3.2247)

4. Gompertz B. 1825 On the nature of the function expressive of the law of human mortality, and on a new mode of determining the value of life contingencies. *Philosophical Transactions of the Royal Society of London* **115**, 513–583. (doi:10.1098/rspl.1815.0271)

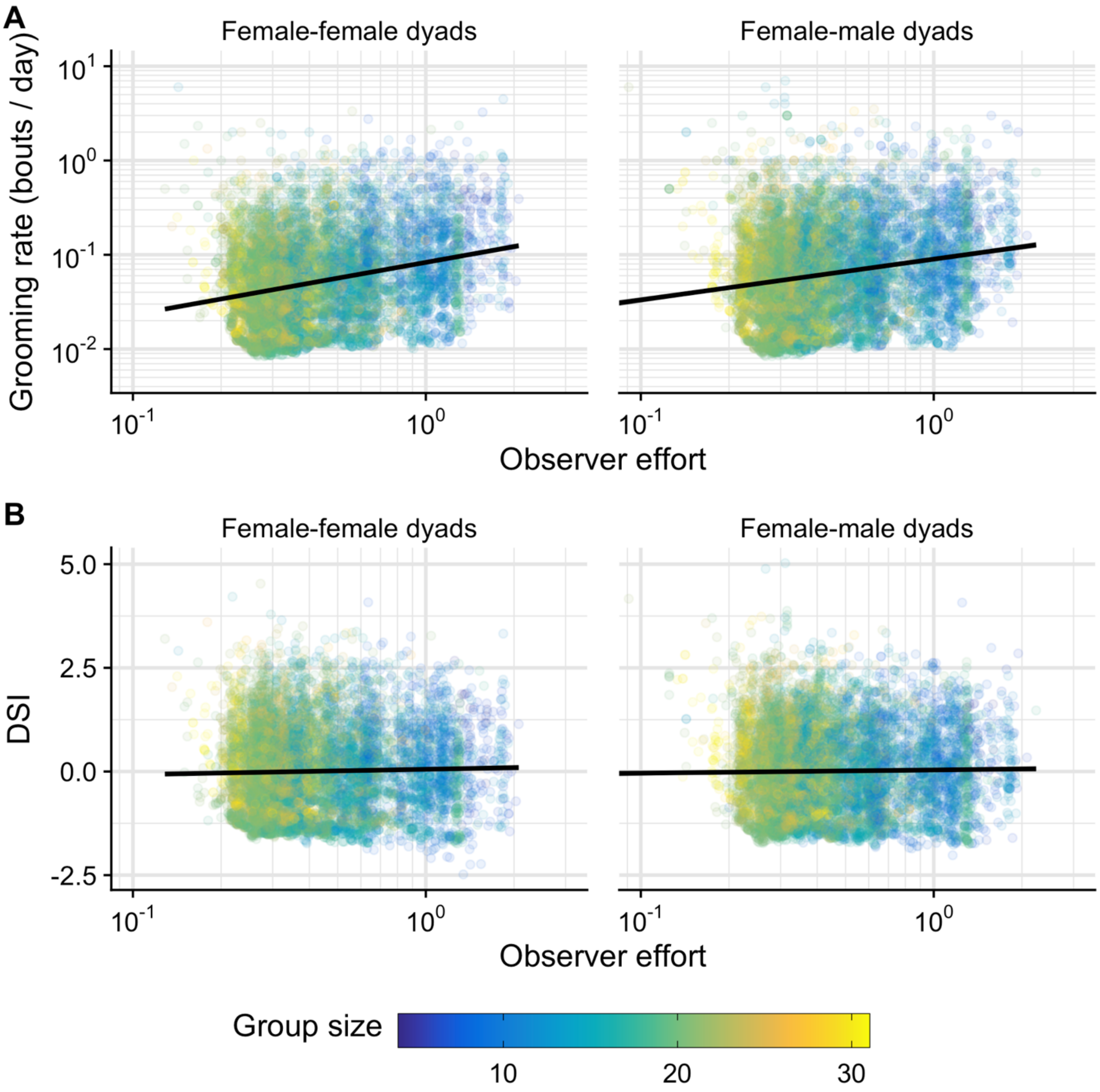

Fig. S1. Correction of bias in grooming rates due to differences in observer effort. A) Relationship between observer effort (focal samples per female per day) and raw grooming rates (observed grooming interactions per day) for 10,000 random dyads of each type, showing that grooming rates generally increase with observer effort. Note that smaller groups (blue colors) are biased toward higher dyadic grooming rates compared to larger groups (yellow colors), largely because observer effort is higher in smaller groups. B) Relationship between the dyadic sociality index (DSI, the residual dyadic grooming rates, standardized by year and dyad type), showing how the bias is removed by regressing dyadic grooming rates against observer effort. For a given level of observer effort, DSI values show similar values regardless of group size.

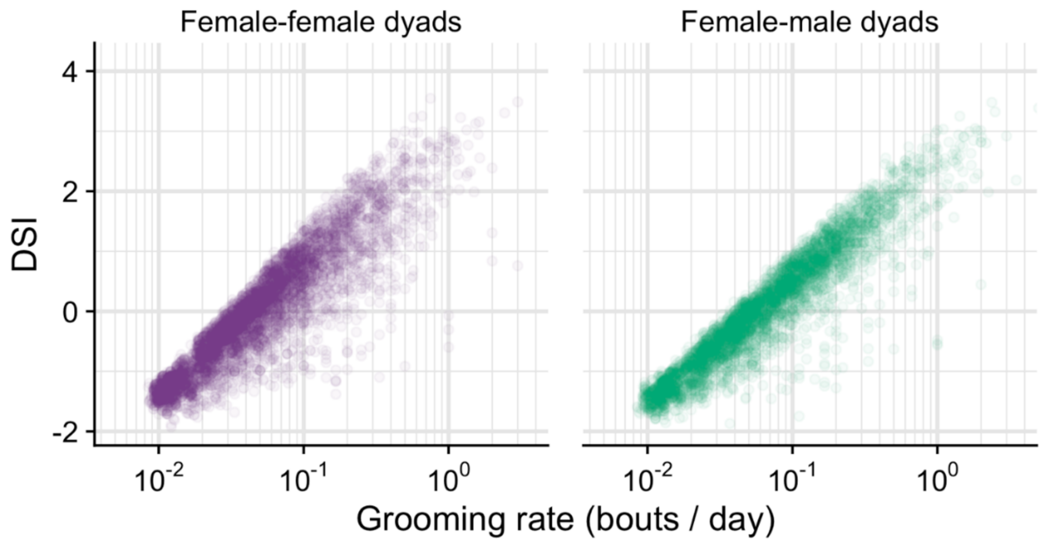

Fig. S2. Relationship between log-transformed raw dyadic grooming rates and DSI values that have been corrected for observer effort (Spearman correlation coefficient = 0.92).

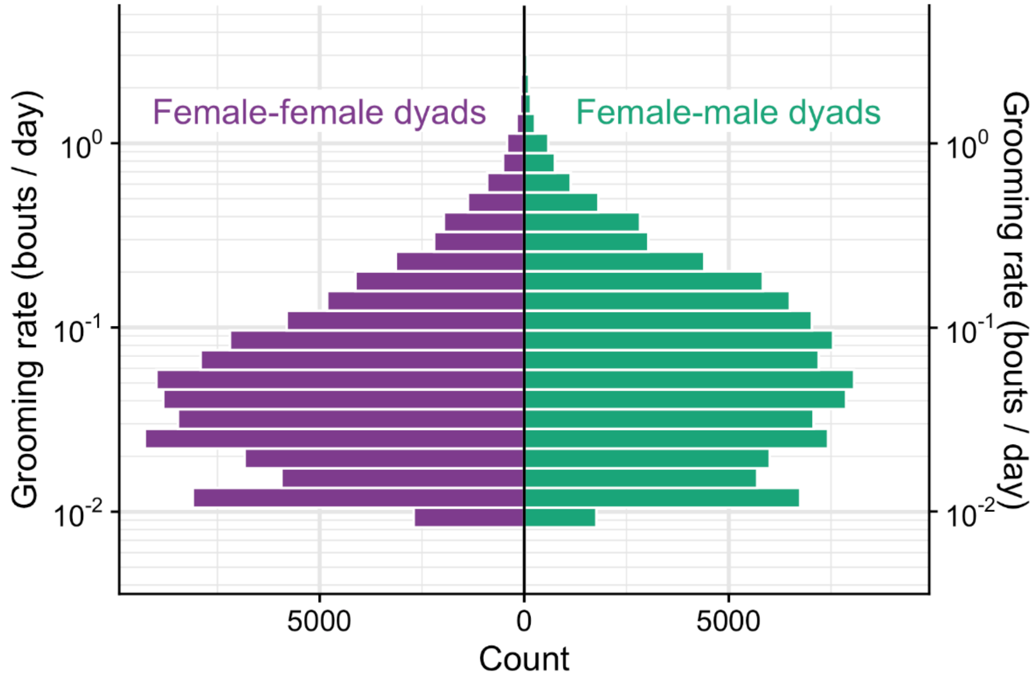

Fig. S3. Comparison of the distributions of dyadic grooming rates for female–female dyads and female–male dyads. The data shown represent 100,000 randomly drawn female-female grooming dyads (purple) and 100,000 randomly drawn female-male grooming dyads (green). The grooming rates used here were calculated as raw frequency of grooming interactions by the dyad per day of observed co-residence during a given year of life.

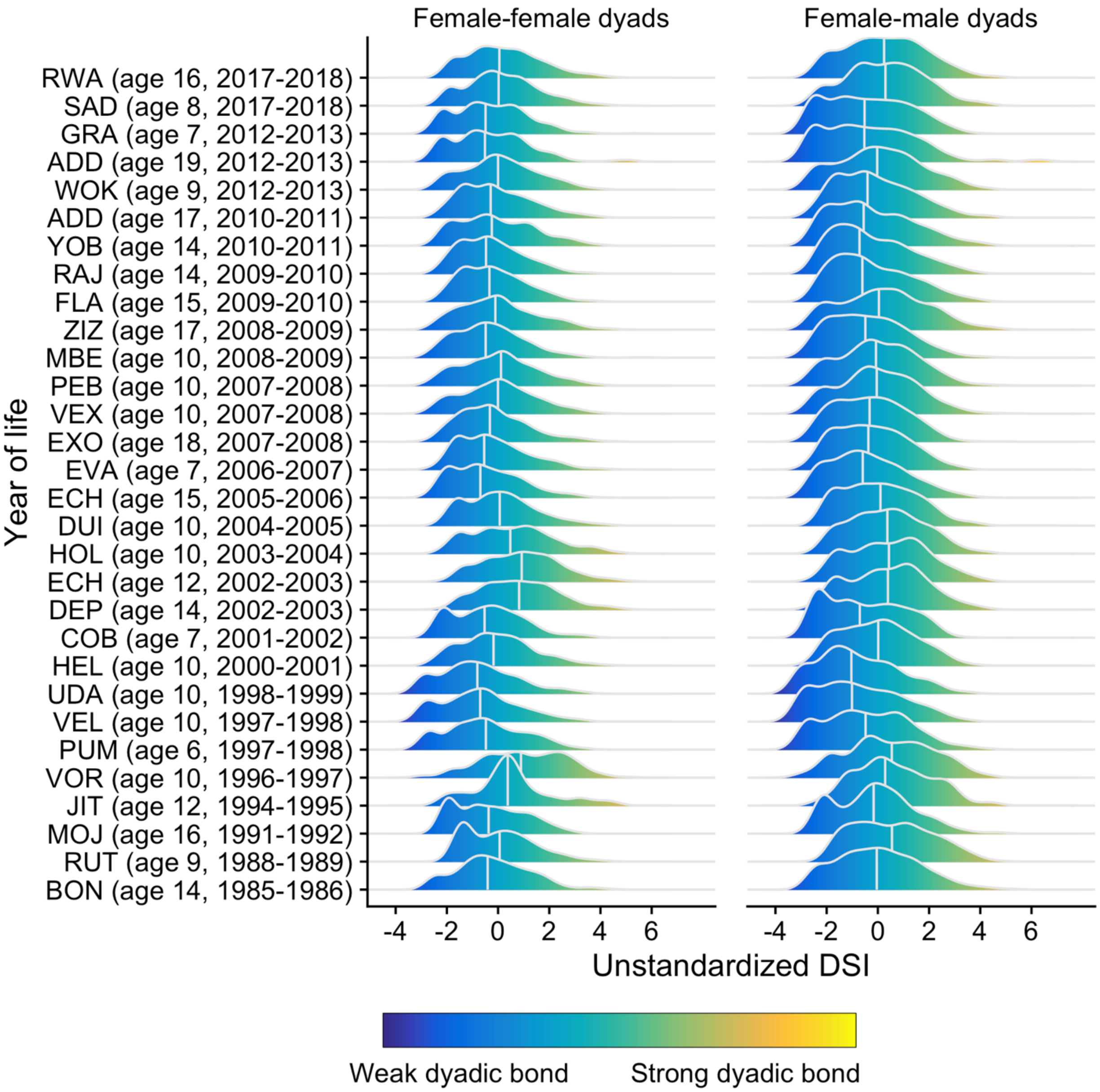

Fig. S4. Dyadic grooming rates corrected for observer effort for the entire population in 30 randomly selected "life-years." Without standardization, the distributions and their median values (white vertical lines) vary from year to year, probably due to environmental and demographic factors. By standardizing within life-years, we removed the effects of population-wide fluctuations in grooming rates that were due to environmental or demographic factors that varied among life years.

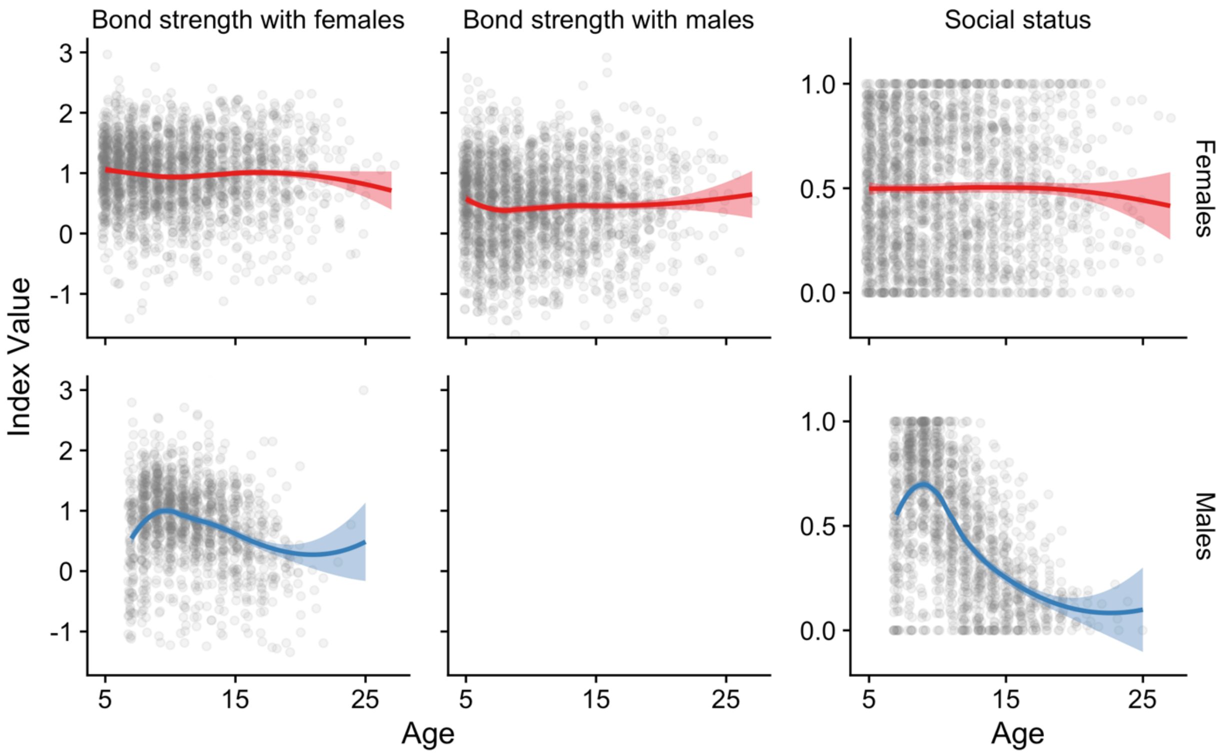

Fig. S5. Relationships between age and raw (unstandardized) values of each covariate in the two sexes. We standardized all covariates by sex and age to account for non-linear relationships between covariates and age, particularly in males, who show peaks in social status and bond strength with females in early adulthood followed by declines in old age. In this way, the covariates used in the mortality model measure deviations from age-typical values for each sex.

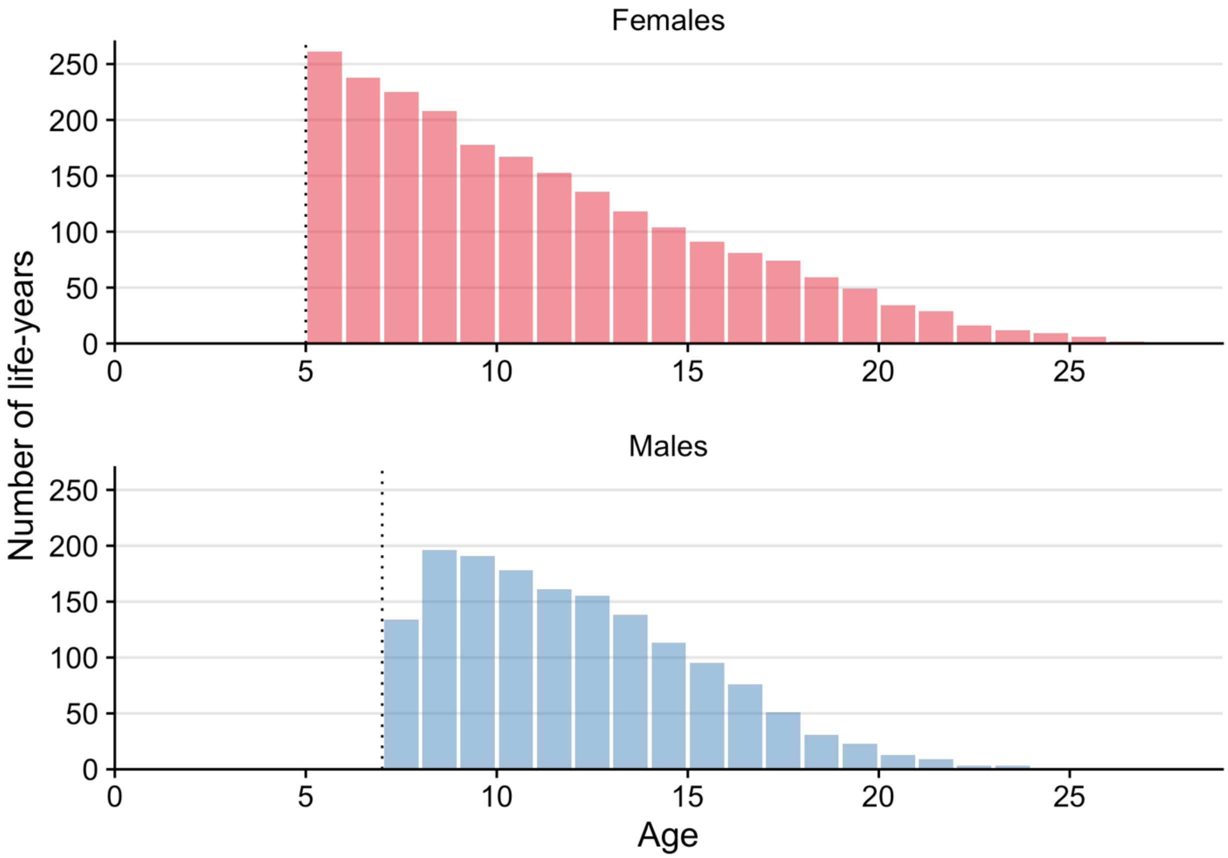

Fig. S6. Approximate number of observed life-years of individuals of each sex and age prior to imputation. The numbers are approximate because, in the case of immigrant males, the ages here represent the best estimates made by experienced observers, but in the model these ages were treated as distributions with associated uncertainties, and therefore the counts could differ in each iteration of the MCMC routine. The vertical dotted lines show age at maturity.

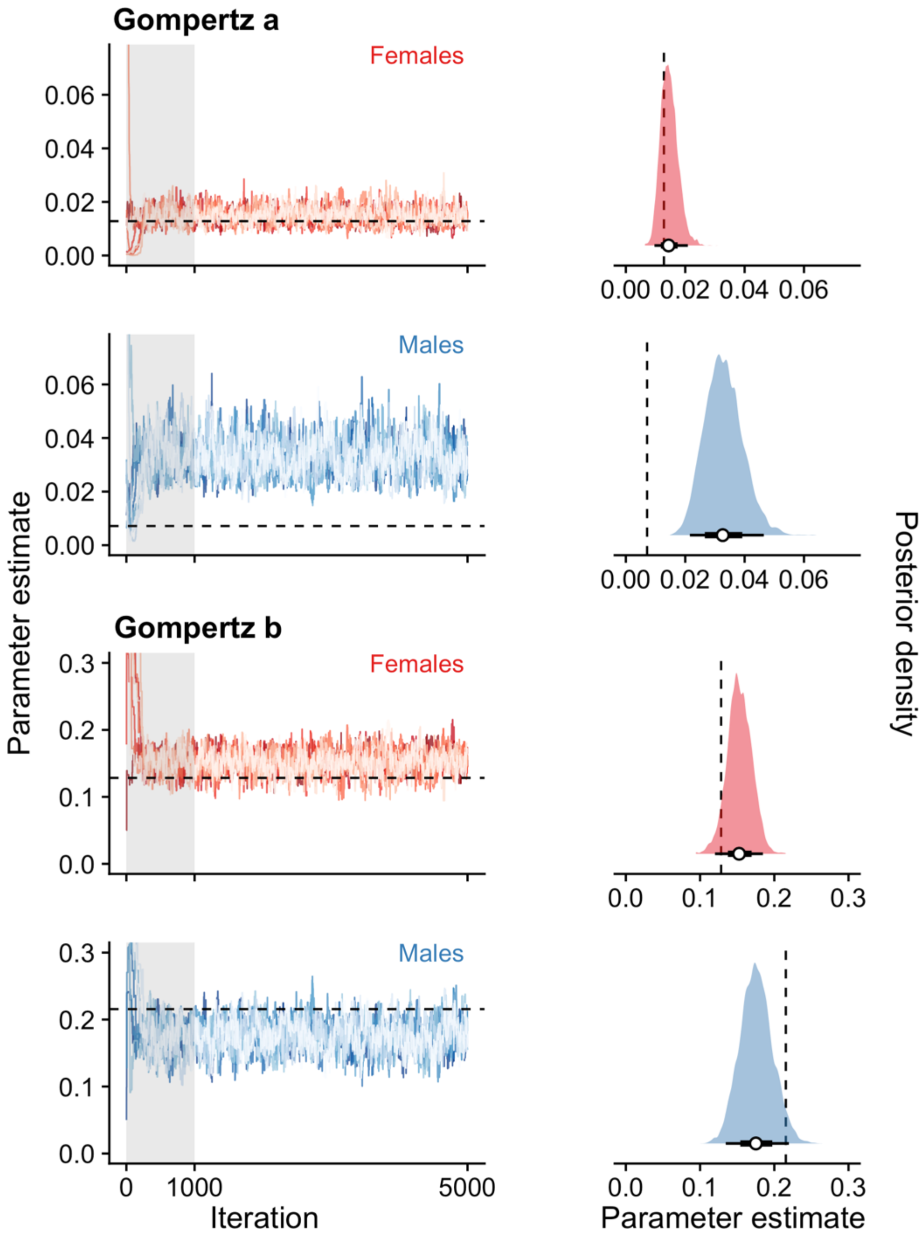

Fig. S7. Diagnostics and summaries of the mortality parameters. The panels at left show traces of the mortality parameter estimates. The eight Markov chains showed good convergence to the same posterior distributions for each of the mortality parameters. The dashed horizontal lines show the priors, and the gray-shaded areas show the initial burn-in sequence of 1000 iterations. The panels at right show posterior densities of the mortality parameters. White points show medians and black bars show 68% and 95% credible intervals of the posterior distributions. The dashed vertical lines show the priors.

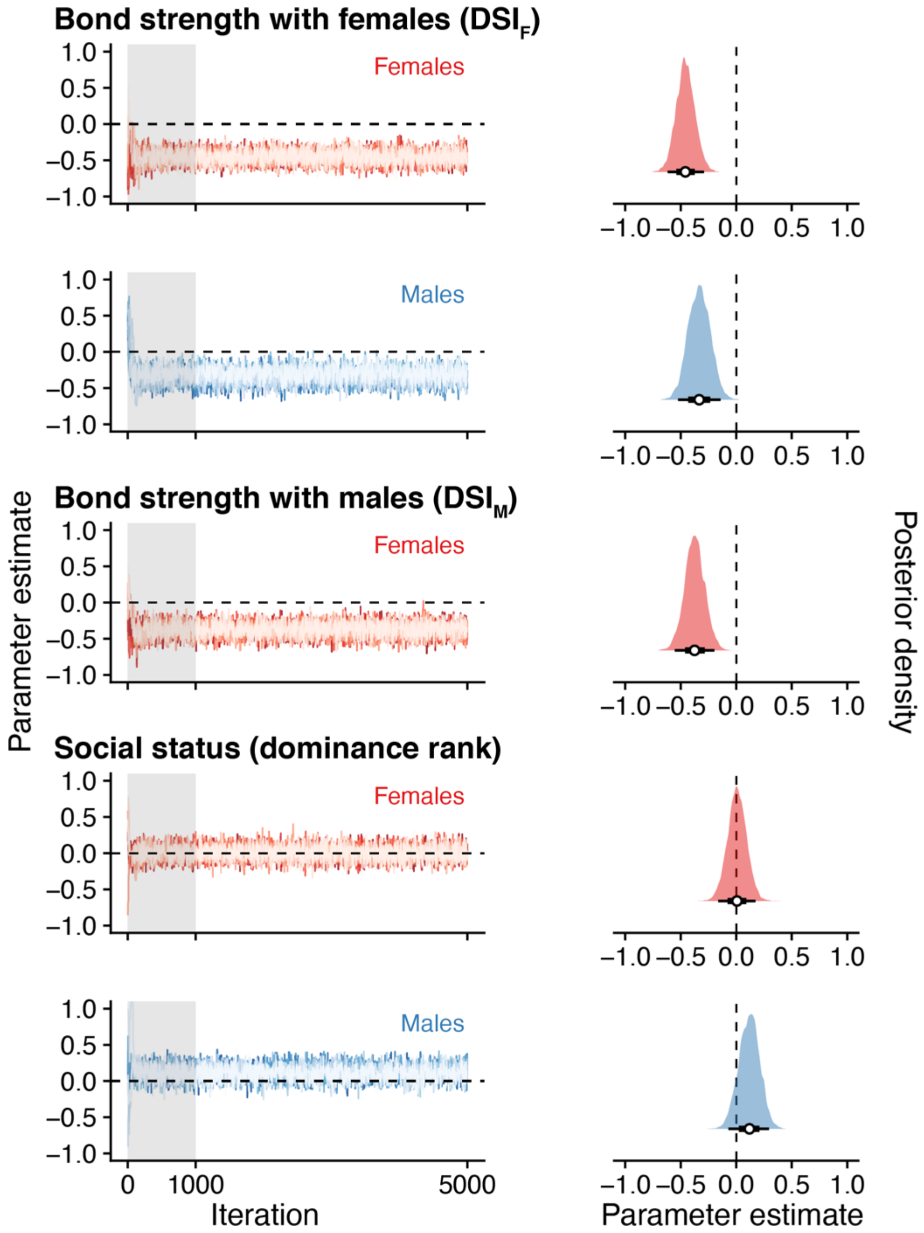

Fig S8. Diagnostics and summaries of the covariate parameters. The panels at left show traces of the time-varying covariate parameter estimates. The eight Markov chains showed good convergence to the same posterior distributions for each of the covariate parameters. The dashed horizontal lines show the priors, and the gray-shaded areas show the initial burn-in sequence of 1000 iterations. The panels at right reiterate Fig. 2 in the main text; they show posterior densities of the time-varying covariate parameters. White points show medians and black bars show 68% and 95% credible intervals of the posterior distributions. The dashed vertical lines show the priors.

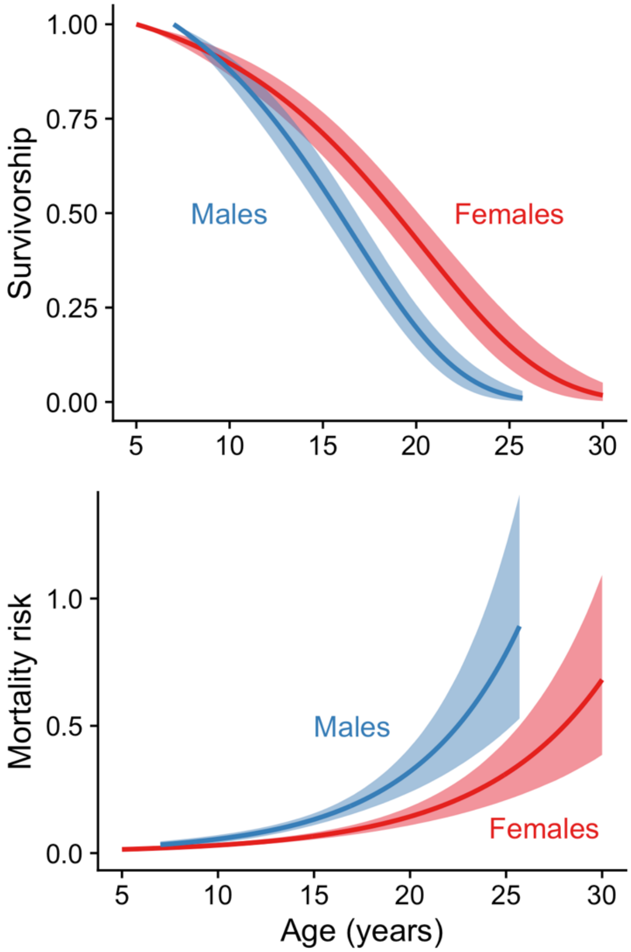

Fig S9. Survivorship and mortality risk for female and male baboons, conditional on reaching age at maturity. These curves correspond to a hypothetical 'average individual' with average values of the three time-varying covariates in all ages.

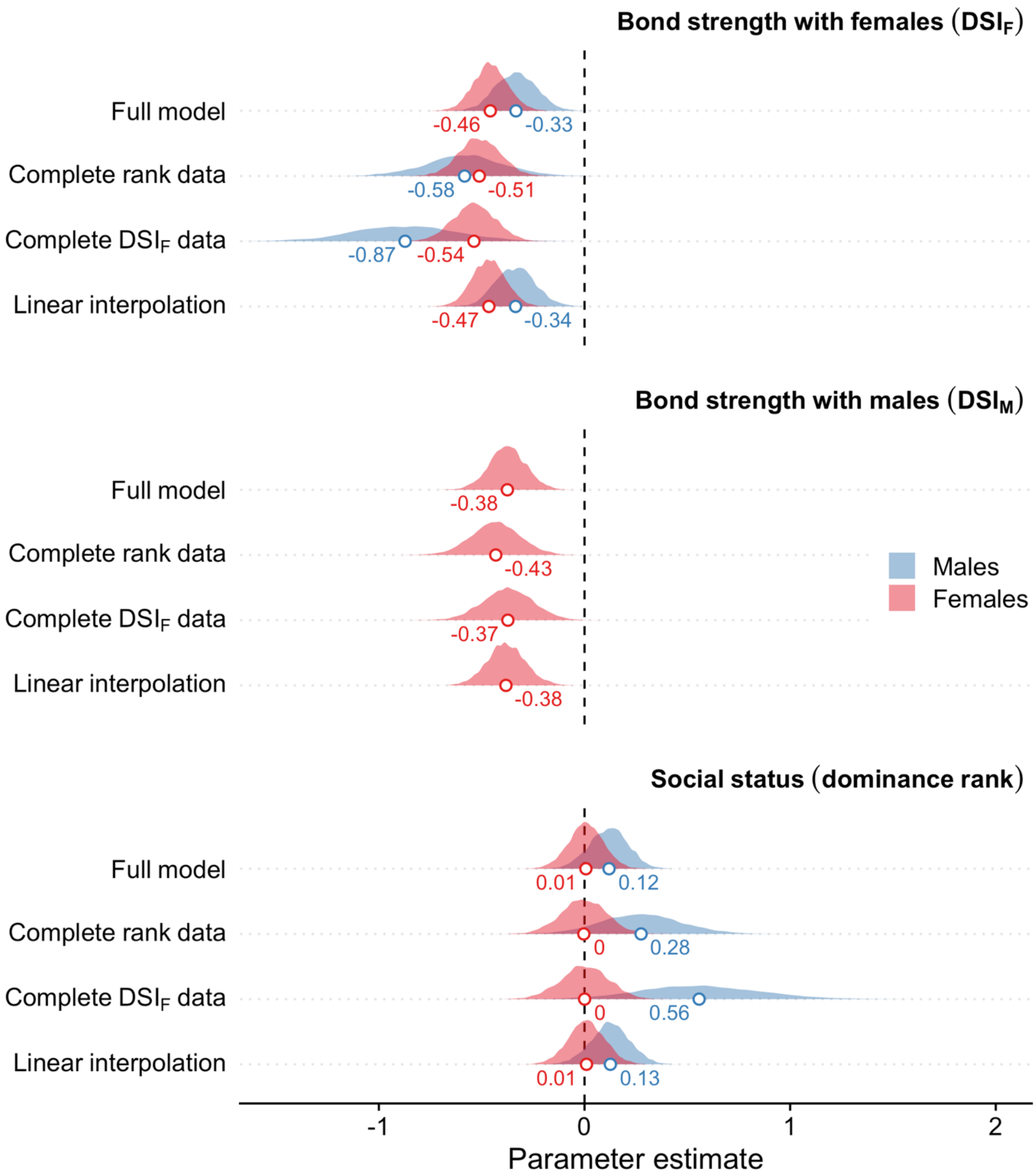

Fig S10. Posterior densities of covariates for the full model (Fig. 2 in main text) and alternate models that use different methods of data imputation. The posterior distributions that result from the linear interpolation method are narrower than in the full model because fewer missing covariates are imputed at each iteration. The posterior distributions that result from complete rank data and complete DSI_F data tests are much wider (especially for social status) due to the reduced sample sizes.

Table S1. Summaries of posterior densities for the mortality parameters and social variables in the model of female and male survival.

Parameter	Sex	Median	Std. Error	68% CI	95% CI	$\hat{R}$
**Gompertz a**	Females	0.014	0.00289	[0.012, 0.017]	[0.010, 0.021]	1.00860
	Males	0.033	0.00636	[0.027, 0.039]	[0.022, 0.046]	1.00359
**Gompertz b**	Females	0.153	0.01644	[0.137, 0.169]	[0.120, 0.185]	1.00843
	Males	0.175	0.02175	[0.154, 0.197]	[0.135, 0.220]	1.00292
**DSI_F**	Females	-0.458	0.08341	[-0.539, -0.374]	[-0.617, -0.290]	1.00183
	Males	-0.333	0.09868	[-0.432, -0.234]	[-0.524, -0.142]	1.04726
**DSI_M**	Females	-0.375	0.08914	[-0.460, -0.284]	[-0.553, -0.197]	1.01110
**Dominance rank**	Females	0.007	0.08522	[-0.077, 0.090]	[-0.162, 0.173]	1.00140
	Males	0.119	0.09357	[0.024, 0.209]	[-0.069, 0.296]	1.00662
